# Texture Image Classification Based on Deep Learning and Wireless Sensor Technology

**DOI:** 10.1155/2022/1761635

**Published:** 2022-05-24

**Authors:** Fengping Chen, Jianhua Qi, Xinquan Li

**Affiliations:** ^1^Department of Mechanical and Electrical Information, Weifang University of Science and Technology, Weifang, Shandong 262700, China; ^2^School of Public Health, Weifang Medical University, Weifang, Shandong 261053, China

## Abstract

The main purpose of the object detection process is to determine the category of the scene object and use the display 3D and 3D frame size. At present, in the case of 3D object detection, we can extract more accurate features by learning a large number of data, and this deep learning network has good results, but there is a very big problem, including the error of input information, extraction error, and so on. Therefore, solving the above problems has become an important direction to promote the rapid development of 3D target detection technology. This paper mainly studies the deep learning wireless sensor technology and also studies the deep learning infrared and visible image fusion. At the same time, based on the introduction of wireless sensor technology and research status, this paper summarizes the existing algorithms. Texture image classification is a more important visual cue in life. Because it will be affected by light intensity, noise size, image scale, and so on. This makes the classification and feature extraction of image scale and texture image more difficult. To solve these problems has become a hot topic of computer vision research in recent years. The shape of the point cloud is completed by using the 3D target detection method to complete the algorithm research. The radar point cloud is extracted by the 3D target detection method, and the radar point group of the overall shape of the object is obtained. The principal component analysis algorithm is used to extract the principal features of the radar point cloud with the complete shape of the object, and the more accurate 3D target frame is obtained after feature adjustment.

## 1. Introduction

Image fusion combines images acquired by multiple sensors to generate images with more information, which makes the images richer and more robust. Using deep learning in computer vision makes the image fusion of deep learning get great development and has excellent performance. In this paper, based on the fusion of infrared and visible light, the multifocus image fusion of deep learning is studied in detail. The main task of this paper is to introduce the background of image fusion and summarize the existing algorithms. Next, the depth based image fusion algorithm is analyzed in detail, and an image fusion algorithm is provided [1]. Two-dimensional target detection technology is more and more mature for the sustainable development of discipline knowledge, but the state of three-dimensional target detection technology is quite different. Three-dimensional target detection technology only adds one dimension on the basis of two-dimensional target detection technology, but the difficulty of implementation increases in the form of geometric multiples. Because the 3D object detection technology is closer to the real world, the 3D target wireless sensor technology is the target that scientists have been looking forward to. At present, with the continuous updating of equipment, the information that researchers can apply to three-dimensional target detection tasks is also expanding, from monocular RGB images taken by color cameras to binocular RGB images, to depth images taken by depth sensors, and then to radar point clouds taken by wireless sensor technology, The information that can be applied to 3D target detection task also presents a trend of diversification [2]. This paper proposes the following methods to solve the problem of texture image classification, using deep convolution neural network to extract and classify images. Next, a total vector representation of the original image is created to collect the function vectors of each cut. Finally, it is classified into existing clusters to solve the problem of texture image classification. Check the effectiveness of 3D reconfiguration and the solution strategy for a specific application. In the 3D reconfiguration system, it is necessary to read the local features of the rotating image and keep the scale and brightness unchanged. Scale invariant function transformation or sift is a computer vision algorithm, which can detect the local features of the image and further interpret it [3]. It searches for extreme points in space and extracts their positions, scales them, and makes them rotation invariant. The features extracted by SIFT are based on some local points of interest, which are independent of image size and rotation. At the same time, it has a strong tolerance to light, noise, and angle error. According to these characteristics, this paper uses sift as a feature point extraction and matching algorithm.

## 2. Related Work

Literature [4] introduces the use of deep convolutional network to classify the texture of the image. First, we cut the picture reasonably and use the convolutional network for the characteristic classification of the cut picture. After that, the cut images obtained by the classification are collected and introduced into the original picture to make it a global vector expression. Finally, the traditional cluster classification method is used to solve the classification problem in the image texture. From the perspective of feature extraction, this paper combines the FMD material texture data set and the MINC2500 scene texture data set with the convolutional network to perform rigorous training on image texture classification for the first time [5]. In ImageNet, the neural network that has been trained is used for feature extraction, and further comparisons are made and the results are obtained. On the basis of this experiment, a neural network trained on ImageNet is used for feature extraction. By simulating the scene of the 3D recovery application, the abovementioned classification process is performed on the collected images [6]. Through the classification of multiple objects and scene classification and the side classification of a single object, the results show that good results have been achieved. At the same time, using PCA to display the classification results after dimensionality reduction, we can intuitively find the presence of impurity photos, which is very useful for impurity photo processing. The literature introduces the three-dimensional target detection method of depth sensor and color camera [7]. First, classify the three-dimensional target detection methods according to two aspects and then elaborate on each method in detail, mainly expounding its advantages and disadvantages, and finally, lead to the various improvement methods of this article. The literature introduces a three-dimensional target detection method combining the attention of the empty-volume mixed field [8]. First, a general algorithm for three-dimensional target detection that combines concentration and voids in mixed domains is introduced. Then, the innovations in this method are explained in detail, then the cost function used in this method is introduced, and the various methods of the method are described in the end. The experiment showed the experimental results, and at the same time the results of the experiment were analyzed in detail, so that the effectiveness and advancement of the method have been further verified. The literature introduces the optimization of 3D target detection by introducing point cloud shape completion [9]. It first introduces the overall framework and process of the 3D target detection optimization method that introduces point cloud shape completion and then introduces the various modules of the method in detail, then introduces the cost function involved in the method, and finally introduces the experimental verification of the method and demonstrates the experimental results to get the effectiveness of the algorithm [10]. The literature introduces the causes of heterogeneous wireless network failures and network performance indicators and proposes a fault detection and diagnosis algorithm based on convolutional neural networks. The algorithm first uses the combination of ReliefF and mutual information to reduce the dimensionality of the network feature parameters, selects the most consistent feature from all the feature combinations, and then preliminarily screens the suspicious faulty cells in the network by calculating the similarity of the time series data distribution, and finally a fault diagnosis model based on convolutional neural network is used to locate the cause of the fault in the suspicious faulty cell [11]. The simulation results show that the algorithm greatly improves the accuracy of fault handling and the delay of fault handling.

## 3. Sensor Wireless Technology and Texture Image Classification Algorithm Model

### 3.1. Sensor Wireless Technology

**Figure 1:**
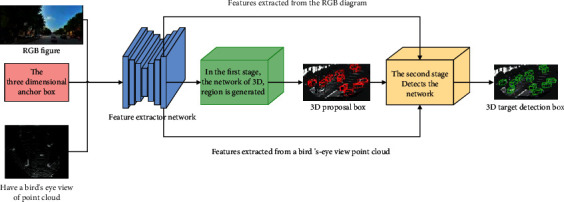
Overall network architecture diagram.

In this paper, a three-dimensional target detection network constructed by mixed-domain attention and spatial convolution is combined. The overall flowchart is shown in Figure 1. The input of the overall network framework includes RGB map and radar point cloud. The network framework is mainly composed of three parts: (1) The network framework part of the feature extractor is constructed to extract the features of various input images; (2) the region generating network part is influenced by Faster R-Inspired by two-stage methods such as CNN, SPP-Net, Fast R-CNN; the first stage network in the network framework uses a region to generate a network to return to the target's three-dimensional proposal frame; (3) the maintenance part of the second-stage network is for more accuracy, to locate and clarify the position on the three-dimensional target of the network regression in the area.

The network as a whole is constructed by the above three parts, but the ultimate goal of the network is to jointly train the network to classify objects and return to its three-dimensional target frame. In the region generation network stage, it is necessary to train the classifier of the network and regress the centroid coordinates of the three-dimensional box of the object and its size. Then, the network uses the “smooth L1 loss” [12] function as a cost function to calculate the center coordinates and the size of the regressed 3D box. The “foreground or background” score regression training is carried out, and the cross entropy function in the classifier is used to calculate the cost function. When calculating the regression loss, it is necessary to ignore the background anchor point frame in the network, calculate the cross ratio between the 3D anchor point frame and the actual frame in the bird's eye view, and mark the 3D frame to determine the background anchor point frame. The second-stage detection network needs to return to the three-dimensional direction of the three-dimensional frame obtained in the first stage to obtain an accurate target three-dimensional frame [13]. The loss function is similar to the region generation network. The direction estimation of 3D target detection frame also uses smooth L1 loss function as the cost function. Therefore, the network as a whole combines the above three cost functions and finally returns to the target category and three-dimensional orientation frame.

The new feature map *μ*(*x*) can be obtained by the following matrix dot product formula (1) as

(1) $$\begin{array}{c} \mu \left(x\right)=W_{\mu }x. \end{array}$$

After the new characteristic map is obtained using the above characteristic conversion operation, the new characteristic map is nonlinearly processed using the Re-LU activation function to perform the response characteristic *μ*(*x*_*i*_) corresponding to the original input characteristic *f*_*b*_ as follows: Corresponding to the characteristic of the new function of *i* mapping, the characteristic response conversion formula is the same as formula (2).

(2) $$\begin{array}{c} e_{i}=\text{max}\left(\mu \left(x_{i}\right),0\right). \end{array}$$

Then, the attention score is obtained based on the acquired characteristics, and the calculation formula is as follows:

(3) $$\begin{array}{c} {\text{score}}_{i}=\frac{\exp\left(e_{i}\right)}{\sum_{i=1}^{L}\text{exp}\left(e_{i}\right)}. \end{array}$$

Finally, the new feature map of the input *f*_*b*_ is multiplied by the attention score to calculate the final output value of the feature map. The calculation formula is as follows:

(4) $$\begin{array}{c} f_{b_{-\alpha ut}}=\sum_{i=1}^{L}{\text{score}}_{i}\mu \left(x_{i}\right). \end{array}$$

By adding the input space consideration mechanism in the feature extraction, the grid can perform geodetic transformation in a more representative area of the image, thereby paying more attention to the actual target of the image area. It helps to improve the accuracy of the 3D target detection process. The calculation formula is as follows:

(5) $$\begin{array}{c} z_{a}=F_{sq}\left(f_{a}\right)=\frac{1}{W_{3}\times H_{3}}\sum_{i=1}^{W_{3}}\sum_{j=1}^{H_{3}}f_{a}\left(i,j\right). \end{array}$$

The calculation formula of excitation *F*_ex_ and *w*_az_ is as follows:

(6) $$\begin{array}{c} s_{a}={\text{F}}_{\text{cx}}\left(z_{a},w\right)\\ =\text{s}\left(\text{q}\left(z_{a},w\right)\right)\\ =\left.\text{s}\left(w_{2}dw_{1}z_{a}\right)\right). \end{array}$$

The process formula is as follows:

(7) $$\begin{array}{c} f_{a_{-}\text{out}}={\text{F}}_{\text{sc}}\left(f_{a},s_{a}\right)=S_{a}\cdot f_{a}. \end{array}$$

**Figure 2:**
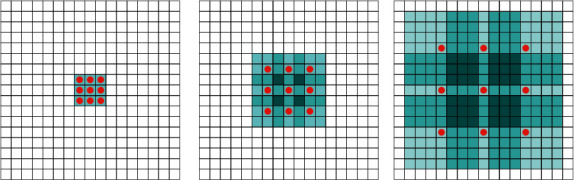
Hole convolution operation with a kernel size of 3 × 3.

It can be seen from Figure 2 that rate is the nonhole pixel distance of the hollow convolution kernel, and it can also be obtained through its structure. The hollow pixels in the kernel have two functions. First, the actual number of pixels in the convolution kernel does not change, so the amount of parameters calculated in the convolution process is actually unchanged. Second, due to the existence of holes, the size of the hole convolution kernel actually becomes larger, which leads to a larger feeling of convolution.

### 3.2. Texture Image Classification Algorithm Model

With the advent of the big data era, deep learning and other technologies have made continuous progress on the original basis. With the help of powerful computing power, people can use complex neural network models to mine and extract key information from massive amounts of data [14]. Especially in a complex heterogeneous network environment, thousands of network nodes will generate a large amount of network operation information every day. The fault diagnosis model based on convolutional neural network proposed in the previous chapter can establish network symptoms and fault roots. The mapping relationship between causes achieved better fault diagnosis accuracy and diagnosis delay, but the actual network operation data recorded by the OAM system may have the problem of missing fault category labels or too general fault category labels; that is, data with valid fault category labels occupies a relatively small proportion of the total data [15]. Therefore, for a supervised deep learning algorithm such as CNN, processing this type of data may not achieve ideal results. Therefore, this chapter proposes an improved method, generating countermeasure network fault diagnosis algorithm.

The texture feature of an image is generally represented by statistical features, and the formula is as follows:

(8) $$\begin{array}{c} \text{ENE}=\sum_{i=0}^{M}\sum_{j=0}^{M}{\left(i,j\right)}^{2}. \end{array}$$

Among them, *M* represents the number of gray levels of the image.

Contrast:

(9) $$\begin{array}{c} \text{CON}=\sum_{i=0}^{M}\sum_{j=0}^{M}{\left(i-j\right)}^{2}p\left(i,j\right). \end{array}$$

Homogeneity:

(10) $$\begin{array}{c} \text{HOM}=\sum_{i=0}^{M}\sum_{j=1}^{M}\frac{p\left(i,j\right)}{1+{\left(i-j\right)}^{2}}. \end{array}$$

Second-order entropy:

(11) $$\begin{array}{c} \text{ENT}=-\sum_{i=0}^{M}\sum_{j=0}^{M}p\left(i,j\right)\text{lg}p\left(i,j\right). \end{array}$$

Relativity:

(12) $$\begin{array}{c} \text{COR}=\sum_{i=11}^{M}\sum_{j=0}^{M}\frac{ljp\left(i,j\right)-\mu_{x}\mu_{y}}{\sigma_{x}^{2}\sigma_{y}^{2}}. \end{array}$$

It is calculated by the following formula:

(13) $$\begin{array}{c} \mu_{x}=\sum_{i=0}^{M}i\sum_{j=0}^{M}p\left(i,j\right), \end{array}$$

(14) $$\begin{array}{c} \mu_{y}=\sum_{i=0}^{M}j\sum_{i=0}^{M}p\left(i,j\right), \end{array}$$

(15) $$\begin{array}{c} \sigma_{x}=\sum_{i=0}^{M}\left(i-\mu_{x}^{2}\right)\sum_{j=11}^{M}p\left(i,j\right), \end{array}$$

(16) $$\begin{array}{c} \sigma_{y}=\sum_{t=1}^{M}\left(i-\mu_{y}^{2}\right)\sum_{j=10}^{M}p\left(i,j\right). \end{array}$$

To use the LBP operator, you first need to set the window, use the selected window as the threshold, and then get two results. A result of 0 means not greater than the threshold, and a value of 1 means greater than or equal to the threshold. In this way, the binary code will be converted to a decimal code so that the LBP value can be obtained clockwise.

The formula is as follows:

(17) $$\begin{array}{c} \text{LBP}\left(x_{c},y_{c}\right)=\sum_{p=0}^{p-1}2^{p}s\left(i_{p}-i_{c}\right). \end{array}$$

*s* is a symbol representing information as follows:

(18) $$\begin{array}{c} s\left(x\right)=\left\{\begin{array}{cc} 1, & \text{if }x\geq 0,\\ 0, & \text{else. } \end{array}\right. \end{array}$$

When learning the basic work of LBP, we will learn how to create local texture features and how the central pixel gradually switches the local area into a binary mode. That is to say, in the local binary mode, there is the invariance of grayscale changes. LBP is very effective in describing local texture characteristics and was inspired by a series of follow-up studies [16]. It has been improved in many ways, including the following: adding noise will not affect the stability of the system, and it will not change the scale of the image. Among them, an improved local binary pattern (ILBP) operator is more discriminative than the existing LBP features. We found that it can explain the texture of the local image. Recently, deep LBP has been proposed, which builds a cascading structure based on the ideas in the field of deep learning, and continuously abstracts the functions of LBP to improve the difference of functions.

So the maximum separation hyperplane becomes the following problem:

(19) $$\begin{array}{c} \underset{w,b}{\text{min}}\frac{1}{2}{\left\|\omega \right\|}^{2},\\ \text{s.t. }y^{\left(i\right)}\left(\omega^{T}x\left(i\right)+b\right)\geq 1,\;i=1,2,\ldots n. \end{array}$$

Find the partial derivative of Ω and *b*, respectively, set it to 0, and find the minimum value:

(20) $$\begin{array}{c} \frac{\partial L\left(\omega ,b,a\right)}{\partial \omega }=\omega -\sum_{i=1}^{n}a_{i}y_{i}x_{i}=0,\\ \frac{\partial L\left(\omega ,b,a\right)}{\partial b}=\sum_{i=1}^{n}a_{i}y_{i}=0. \end{array}$$

It can be derived from (15):

(21) $$\begin{array}{c} \omega =\sum_{i=1}^{n}a_{i}y_{i}x_{i}. \end{array}$$

By incorporating (16) and (17) into (14), then:

(22) $$\begin{array}{c} L\left(\omega ,b,a\right)=\sum_{i=1}^{n}a_{i}-\frac{1}{2}\sum_{i,j=1}^{n}a_{i}a_{j}y_{i}y_{j}x_{i}^{T}x_{j}=0. \end{array}$$

Plus the above restrictions:

(23) $$\begin{array}{c} \left\{\begin{array}{c} \text{s.t. }a_{i}\geq 0,\;i=1,2,\ldots ,n,\\ \sum_{i=1}^{n}a_{i}y_{i}=0. \end{array}\right. \end{array}$$

The final decision function is

(24) $$\begin{array}{c} f\left(x\right)=\text{sgn}\left(\sum_{i=1}^{n}y_{i}a_{i}\left(x_{i}\cdot x\right)+b_{0}\right). \end{array}$$

## 4. Application of Deep Learning and Sensor Wireless Technology in Texture Image Classification Method

### 4.1. Texture Image Architecture

In recent years, with the continuous improvement of people's living standards and the continuous progress of smart mobile devices, the contradiction between people's demand for various data services and emerging applications and the capacity that the current network can carry has become increasingly prominent. As one of the key technologies for expanding the current network system capacity and coping with the blowout growth of mobile data traffic, this technology has gradually received extensive attention and research from academia and industry. The improvement of computer computing power has also promoted the development of the field of deep learning, thus realizing the rapid mining of useful information in massive data. It mainly introduces the architecture and problems of heterogeneous network, as well as the existing network fault diagnosis technology and the deep learning algorithm used in this article.

**Figure 3:**
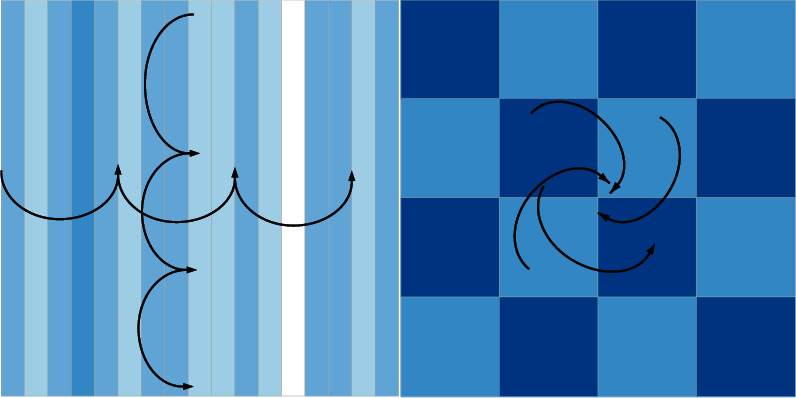
Schematic diagram of texture structure.

The texture image contains many repetitive features that conform to the statistical properties. As shown in Figure 3, the local texture information will be redisplayed in some areas, allowing us to efficiently learn local features, while LSM can store long-term data and learn specific correlations in a larger neighborhood space [17]. In this section, we recommend using LSM units and conversion levels to show the power of textures.

The features extracted from the existing 3D target detection network lack dependency on the regional channel and cannot be expanded without loss of resolution, so there are some error problems when returning to the 3D frame. Among them, the feature extractor of the network is designed as a pyramid network structure to extract high-resolution feature maps; at the same time, the feature extractor used in the network, the input layer of the convolutional neural network, combines a spatial domain attention mechanism, to intensify the target features in the region and weaken the background features by spatially transforming the image; and the feature extractor used in the first four convolution stages of the convolutional neural network is the encoder of the convolutional neural network. In the network stage, a channel domain attention mechanism is combined so that the network can learn the weight of each channel independently to obtain more important channel features; finally, combine the hole convolution and attention mechanism before the output layer of the convolutional neural network and get the final key feature.

### 4.2. Data Set and Parameter Setting

We use two sets of texture data for testing. The first description is that the texture data set (dtd) has 47 categories, with a total of 5640 images per 120 images.

1. The threefold cross-validation method conducts mutual verification experiments and reports the average index and standard deviation. The data is randomly divided into three independent parts, two of which will be used for training and the other for testing.
2. Experiment with tenfold cross-validation, and report the average index and standard deviation. The data is randomly divided into 10 separate parts, 9 of which are used for training and the rest are used for testing.

### 4.3. Data Set Training Results

The FMD texture data set is divided into 8 categories, and each category contains 90 images. It turns out that the amount of data is very small, and in order to expand the texture data set, each graphic basically covers the entire picture with texture information, so each graphic is divided into 9 equal parts. The data set expanded in this way has 810 images, and this is used as the training data set. Extract 90 of each category into the test data set in the extended data set.

**Figure 4:**
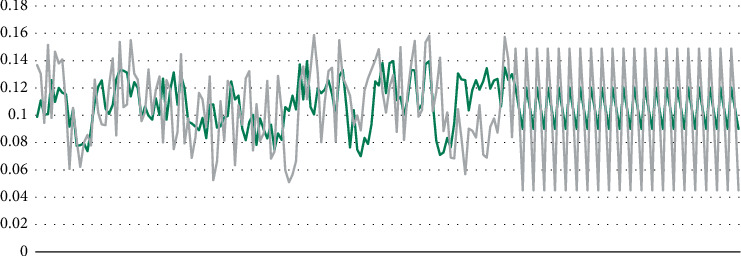
FMD data set training results.

The IDE used in this experiment is Pycharm based on the Tensorflow deep learning framework. The value of hacksprule is set to 1*e* − 3, and the training rounds are 45 and 95, respectively. The training batch size was tested in two cases of 55 and 85, respectively. In either case of 85, the tested batch size was set to 8, and the inefficiencies were 0.1 and 0.4, respectively. Using other parameters to train the neural network, the experimental results are similar. The experimental results are shown in Figure 4.

The MINC2500 texture data set is divided into 21 categories. There are a total of 56,000 photos in the data set document, but more than 20,000 photos have been downloaded, and there are few categories of photos, and the least category is less than 90 photos. The categories with less than 400 images were deleted, and finally 7 categories were set up, with an average of 1900 photos in each category, and this was used as the training data set. Extract 90 images from each category to the test data set.

**Figure 5:**
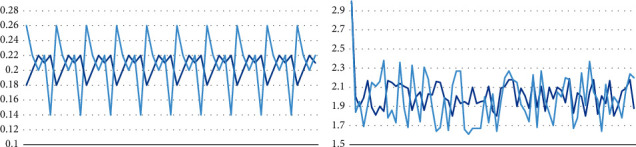
MINC data set training results.

The IDE used in this experiment is Pycharm, which is based on the Tensorflow deep learning framework and selects the AlexNet neural network model. The experimental results are shown in Figure 5.

### 4.4. Simulation of Experimental Results

First, we created this method with classic functions (GLCM, LBP) and low classifiers (SVM, RF). As shown in Table 1, LBP shows the best results of general classification methods, and its accuracy is 28.43% higher than traditional methods. It can be seen that the learning effect is better by using deep convolutional neural network transmission. This also proves the powerful expressive ability of deep neural networks.

Table 1 shows that, using LSM and CNN network structures to improve the data, the results of ResNet50 classification can be improved by up to 5%. The accuracy of classification can be improved by more than 0.59%.  Table 2 uses all the data in the training set and the training in the validation set as the center. In the lowest model selection set, 90% of the verified data is used. The DTD file uses the 3-fold cross-validation test, and the FMD uses it. 10-fold cross-validation test is used to improve the final classification accuracy of training data and network reorganization DTD.

Table 2 shows that using ResNet50 as DTD increases the average accuracy of the hidden layer by 72.99% and increases the rate of hidden layer coding by 2.24%. As shown in the last row of Table 2, both data sets have reached a high level. These results confirm that adding hidden codes by converting the long-range relative function into a new embedding area can improve the attribute separation and improve the results.

On the other hand, deep neural network needs a lot of MKS. On the other hand, deep neural network can learn and share basic functions and use them in different texture data image segmentation. This section focuses on the joint training of DTD and MKS data sets and discusses the ability to process multiple data simultaneously in the same network, so that we can train DTD and MKS at the same time.

The results of cooperative training using multibranch model are slightly inferior to that of individual models, but the trend of the two data sets is the same, and there is only a small difference between other structures. This shows that cooperative training can effectively use the shared functions learned in neural network to manipulate different data sets. By modifying the fully connected Model-C, we can get good results on both data sets. Therefore, it can be reused in multiple data set models by using less parameters and computation from multiple data sets, as shown in Table 3.

Due to the limitation of memory size, adding improved samples of the same image in different directions in minibatch will reduce the effective processing size of minibatch, that is, the number of other images contained in the minibatch. The results of the DTD in the Ablation experiment are shown in Table 4. As the direction of rotation increases, the classification accuracy tends to increase first and then decrease. On the one hand, increasing the direction of rotation can improve the ability to express functions and improve the classification accuracy and function coding in multiple directions in the same image. On the other hand, reducing the effective minibatch size will make the statistical data unstable. The effect of training is affected and the classification accuracy is reduced.

We can see that the accuracy of the DTD data set has improved from 66.5% to 76.4%. The accuracy of the methods in this chapter has also been greatly improved. The latest FASON method can combine primary and secondary statistical information at the same time to learn more powerful feature expressions. With further improvement to the B-CNN method, the latter is only used for statistical information. However, the results obtained in this chapter are still much better than those of FASON, increasing the absolute accuracy by 3.5%. The results are shown in Table 5.

The above experimental results are similar to the results of the FMD data set. The results are shown in Table 6.

In this chapter, we discussed convolutional neural networks (CNN) and deep neural networks based on long and short-term memory modules (LSTM). These structures can effectively model the texture image chapter/short-distance spatial correlation combined with hidden layer. Learning stronger feature representations can improve the accuracy of texture image classification. First, the multilayer convolutional neural network is used as the basic network to extract basic functions, build a multidirectional long-term storage unit, model the remote texture correlation in multiple directions, and finally hide it. In order to achieve the final classification, the function has also been coded and converted. Experiments on the two main data sets FMD and DTD show that the method in this chapter has higher classification accuracy than traditional methods. At the same time, the collaborative training of multiple data sets and the comparative study of multidirectional attribute coding are carried out. The experimental results show that the method proposed in this chapter is easy to use and has the potential for further improvement.

## 5. Conclusion

In the real physical world, humans can use their eyes to form stereo vision and recognize various information; in the computer world, we use the same information obtained by the eyes and perform a similar recognition process. Depth sensors require 3D information and color, shape, texture, and other information obtained from color cameras. Therefore, 3D target detection processing based on depth sensors and color cameras has also become a hot spot. This article provides methods for realizing 3D target detection and processing and analyzes in detail two aspects: application data and feature extraction methods. Considering the advantages and disadvantages of each method, we conclude that the two-step 3D target detection method based on depth sensor and color camera is more advantageous in achieving high detection accuracy in 3D target detection processing, and we propose a fault detection based on CNN and diagnostic algorithms. The algorithm first combines information with ReliefF to reduce the dimensionality of the parameters in the network function, selects the best combination of functions, calculates the similarity between the time series data distribution and the network, prescreens suspicious units, and finally passes convolution. The neural network fault diagnosis model of the library finds the cause of the suspicious unit. The simulation results show that the algorithm can significantly improve the accuracy of error diagnosis and the delay of diagnosis.

## Figures and Tables

**Table 1:** Use different basic network structures to reproduce the results of classic methods and deep CNN methods in the DTD data set.

Method	Accuracy (%)
GLCM + SVM	10.26
LBP + SVM	23.69
LBP + RF	28.43
AlexNet	28.72
ResNet50	60.64
ResNet50 (Aug)	65.74
ResNet50-LSTM (Aug)	66.33

**Table 2:** The experimental results of the hidden layer coding method on the DTD (3-fold) and FMD (10-fold) data sets.

Data set	Method	Accuracy (%)
DTD	ResNet50-LSTM (Aug: *T* + *V*)	70.75
	ResNet50-LSTM-Conv-E	72.99

FMD	ResNet50-LSTM (Aug: *T* + *V*)	80.30
	ResNet50-LSTM-Conv-E	80.9

**Table 3:** Comparison of joint training methods.

Joint training method	DTD (%)	FMD (%)	Average (%)
Model-base	75	80.9	78
Model-C	73.34	79.45	76
Model-EC	72.55	80.7	77
Model-LSTM-EC	73.6	79.4	77
Model-C^*∗*^	74.22	75.3	74.76

**Table 4:** The 10-fold experimental result of the multidirectional feature coding model on the DTD data set.

Model	Accuracy (%)
Model-base-bl 6R1	75
Model-base-B8R2	76.6
Model-base-B5R3	74.82
Model-base-B4R4	71.28

**Table 5:** Comparison between the method proposed on the DTD data set and some advanced methods.

Method	Accuracy (%)
DecAF + IFV	66.40
FV-CNN (*s* = 1)	67.70
B-CNN (*s* = 1)	69.50
FASON (Conv5)	72.40
FASON (Conv4 + Conv5)	72.80
Methods mentioned in this chapter	76.50

**Table 6:** Comparison between the method proposed on the FMD data set and some advanced methods.

Method	Accuracy (%)
DecAF + IFV	65.7
FV-CNN (*s* = *l*)	75.3
B-CNN (*s* = *l*)	77.6
DeepTen (sT)	80.4
Methods mentioned in this chapter	80.7

## Data Availability

The datasets used and analyzed during the current study are available from the corresponding author upon reasonable request.

## References

[1] Mandhouj I., Amiri H., Maussang F., Solaiman B. Sonar image processing for underwater object detection based on high resolution system.

[2] Mehta D., Sridhar S., Sotnychenko O. (2017). VNect: real-time 3D human pose estimation with a single RGB camera. *ACM Transactions on Graphics*.

[3] Weng L.-Y., Li M., Gong Z. B., Ma S. G. Underwater object detection and localization based on multi-beam sonar image processing.

[4] Lucas A., Iliadis M., Molina R., Katsaggelos A. K. (2018). Using deep neural networks for inverse problems in imaging: beyond analytical methods. *IEEE Signal Processing Magazine*.

[5] Wang X., Tao Q., Wang L., Li D., Zhang M. Deep convolutional architecture for natural image denoising.

[6] Ledig C., Theis L., Huszár F. Photo-realistic single image super-resolution using a generative adversarial network.

[7] Sárándi I., Linder T., Arras K. O., Leibe B. (2020). Metrabs: metric-scale truncation-robust heatmaps for absolute 3d human pose estimation. *IEEE Transactions on Biometrics, Behavior, and Identity Science*.

[8] Ershadi-Nasab S., Noury E., Kasaei S., Sanaei E. (2018). Multiple human 3d pose estimation from multiview images. *Multimedia Tools and Applications*.

[9] Gu R., Wang G., Jiang Z., Hwang J. N. (2019). Multi-person hierarchical 3d pose estimation in natural videos. *IEEE Transactions on Circuits and Systems for Video Technology*.

[10] Azhand A., Rabe S., Müller S., Sattler I., Heimann-Steinert A. (2021). Algorithm based on one monocular video delivers highly valid and reliable gait parameters. *Scientific Reports*.

[11] McNally W., Wong A., McPhee J. (2018). Action recognition using deep convolutional neural networks and compressed spatio-temporal pose encodings. *Journal of Computational Vision and Imaging Systems*.

[12] Zhao H., Gallo O., Frosio I., Kautz J. (2016). Loss functions for image restoration with neural networks. *IEEE Transcation on Computer Imaging*.

[13] Rocco I., Arandjelovic R., Sivic J. (2019). Convolutional neural network architecture for geometric matching. *IEEE Transactions on Pattern Analysis and Machine Intelligence*.

[14] Gu J., Wang Z., Kuen J. (2018). Recent advances in convolutional neural networks. *Pattern Recognition*.

[15] Johnson J., Alahi A., Fei-Fei Li (2016). Perceptual losses for real-time style transfer and super-resolution. http://arxiv.org/abs/1603.08155.

[16] Guo Z., Zhang L., Zhang D., Mou X. Hierarchical multiscale LBP for face and palmprint recognition.

[17] Zhang S., Liu Y., Li X., Bi G. (2017). Variational Bayesian sparse signal recovery with LSM prior. *IEEE Access*.

